# Editorial: Key Players in Systemic Sclerosis: The Immune System and Beyond

**DOI:** 10.3389/fimmu.2021.770419

**Published:** 2021-09-30

**Authors:** Philippe Guilpain, Danièle Noël, Jérôme Avouac

**Affiliations:** ^1^ Department of Internal Medicine and Multi-Organic Diseases, Local Referral Center for Systemic and Autoimmune Diseases, St Eloi Hospital, Montpellier, France; ^2^ Faculty of Medicine, University of Montpellier, Montpellier, France; ^3^ Inserm U1183, Institute for Regenerative Medicine and Biotherapy (IRMB), St Eloi Hospital, Montpellier, France; ^4^ Department of Rheumatology, Cochin Hospital, AP-HP.CUP, Paris, France; ^5^ Faculty of Medicine, University of Paris, Université Paris Descartes, Paris, France; ^6^ INSERM U1016 and CNRS UMR8104, Cochin Institute, Paris, France

**Keywords:** systemic sclerosis, pathophysiology, co-stimulation, abatacept, innate immune system, mesenchymal stromal cell, extracellular vesicles, cancer

In these times of Covid-19 pandemic, during which medical research is mobilized in the fight against the coronavirus, patients continue to suffer from other acute and chronic diseases and research on them must continue. Systemic sclerosis (SSc) is one of these diseases, both chronic and complex, whose evolution can be fatal and whose clinical repercussions, variable from one patient to another, can result in a profound alteration of autonomy and quality of life. The current crisis of the coronavirus should not make us forget this reality.

At this point, we must recall the well-known generalities about this disease (1). Systemic Sclerosis is a rare multifaceted disease, whose development is related to the interplay of three major pathophysiological mechanisms, *i.e.* vascular impairment, autoimmunity and uncontrolled fibrogenesis. These mechanisms lead to the development of the main disease-related manifestations including skin, lung, heart, and gastrointestinal tract involvement. Fibrosis primarily affects the skin dermis. However, the lungs, heart, and/or digestive tract may also be involved. Vascular impairment leads to the development of Raynaud’s phenomenon, telangiectasia, digital ulcers, pulmonary arterial hypertension and/or vascular renal crisis. These features render SSc a very peculiar autoimmune disorder. Autoimmunity in SSc is mediated *via* the immune cell activation and production of autoantibodies (Abs). The latter are mainly directed against three target autoantigens, namely Topoisomerase 1 (anti-SCL70 Abs), CENP-A/B (anti-centromere Abs) or RNA-polymerase III. Sustained immune and inflammatory stimulation promote pathological fibroblast activation, which results in excessive extracellular matrix protein production and accumulation in tissues.

The clinical presentation of the disease is heterogenous. SSc may present in three classical forms, including limited cutaneous, diffuse cutaneous, and ‘sine scleroderma’ subsets, and may associate with other connective tissue diseases. SSc has a poor prognosis, globally with a severe vital prognosis and potential long-term disability. Its treatment is mainly palliative and based on symptomatic therapies, vasodilators, immunosuppressants, targeted therapies and antifibrotic drugs. Therefore, advances in our understanding of the crucial actors and mechanisms that underlie this disease are essential for improving therapy (hopefully, through the development of innovative therapeutic approaches) and patient outcomes.

In fact, a myriad of questions regarding SSc pathogenesis still remain unresolved. By exploring the most recent advances and discoveries in this field, this Research Topic aimed to transition our understanding of SSc pathogenesis from its old conception (based on the three main mechanisms, *i.e.* autoimmunity, fibrogenesis and vascular involvement) to a modern view.

When we proposed this topic to Frontiers in Immunology (https://www.frontiersin.org/research-topics/7231/key-players-in-systemic-sclerosis-the-immune-system-and-beyond#overview), the most recent advances in SSc had included the following key points (2): (i) fibroblast activation and endothelial impairment are likely dependent on environmental, genetic and epigenetic factors, with a specific contribution of endogenous and/or exogenous oxidative stress; (ii) a link was recently established between cancer and some specific forms of SSc [with particular autoantibodies (i.e. anti-RNA polymerase III and related Abs)], illustrating that the immune response could be directed against malignant targets; (iii) metabolic pathways (such as those implicating PPAR-γ) were also likely important for SSc development and could represent innovative therapeutic targets; (iv) while autologous hematopoietic stem cell transplantation following intensive immunosuppressant therapy had convinced more and more SSc specialists of its interest, progenitor cell-based therapies [such those using mesenchymal stromal cells (MSC)] were under development with great promises; (v) additionally, several pharmacological approaches were also under development to counteract fibrosis and vascular impairment in SSc.

The present Research Topic is entitled “Key players in Systemic Sclerosis - The immune system and beyond…”, since key players in SSc are not limited to the immune system. This is one of the most original specificities of the disease. So, in the present Research Topic, we proposed 20 articles (including 10 review articles and 10 original articles) in the field of SSc, focusing on various cellular, molecular and environmental actors involved in SSc, in relationship with the immune system, and on innovative therapies for SSc. So, you can read in the present book, two original articles on Abs [anti-Ro (Gkoutzourelas et al.), and antiphospholipid antibodies (Sobanski et al.)], illustrating the variety of Ab repertoire in SSc, and discussing the capacity of SSc for overlapping. Herein, you can also find articles on the role of immune system and its interaction with other key players (such as fibroblasts and endothelial cells): the role of the innate immune system (Bhattacharyya et al.; Laurent et al.), the costimulatory pathways (Boleto et al.), the Regulatory T Cells (Frantz et al.), the microchimerism (Di Cristofaro et al.), the relationships between inflammatory cytokines and fibroblasts (Dufour et al.), the induction of endothelial microparticles release by natural killer cells (Benyamine et al.). Otherwise, other key players are presented: the central role of myofibroblasts is extensively reviewed (van Caam et al.), while a novel resistance mechanism to apoptosis is described in fibroblasts and vascular smooth muscle cells (Takata et al.); the deleterious effects of oxidative stress is discussed considering endothelial-to-mesenchymal transition (Thuan et al.), whereas the beneficial role of Nrf2-antioxidant response is illustrated in the murine model of HOCl-induced scleroderma (Kavian et al.; Kavian et al.). The intriguing relationships between SSc and cancer are also presented in a review article (Maria et al.), underlining the similarities between fibrosis and cancer development as well as the most recent data on paraneoplastic forms of SSc. The phenomenon of vascular leaking is also presented as a key player in the early phase of SSc (Bruni et al.), and the stages of fibrosis progression are now documented in the murine HOCl-induced model (Maria et al.). Finally, therapeutic approaches based on autologous hematopoietic stem cell transplantation (Del Papa et al.) or MSC (Maria et al.; Peltzer et al.; Maria et al.; Rozier et al.) are also presented in this book. We thank all the authors for their contribution.

Three years and one pandemic later, the content of this book remains fully relevant and continues to shed light on new ways of understanding and treating SSc. We would now like to highlight some of the data and concepts described in this book, and discuss them in light of other recent studies.

We encourage the reading of the review article on the innate immune system (Laurent et al.). While macrophages and type I interferon have been considered for many years as key players in SSc, several other components of the innate immune system (such as pathogen-recognition receptors, platelet-derived danger-associated molecular patterns, innate lymphoid cells, and plasmacytoid dendritic cells) exhibit now an emerging role. Research on the subject is ongoing and promising. A recent study (3) demonstrates the role of innate lymphoid cells-2 (ILC2) both in human and murine SSc, and more precisely the interplay between these cells and cytokines (namely, TGFβ and IL10) in the development of fibrosis. Interestingly, TGFβ could induce the switch of ILC2 from an ‘inflammatory’ phenotype (KLRG1^high^) to a ‘natural’ phenotype (KLRG1^low^), which exhibits a more potent capacity for fibrogenesis. Even more interesting, pharmacological TGFβ blockade with the anti-fibrotic agent pirfenidone did not significantly reduce the fibrotic process, while a combined treatment with pirfenidone and IL10 did. This therapeutic response was associated with a reduced number of skin infiltrating ILC2 and an enhanced expression of KLRG1. These findings illustrate the complexity of the disease as well as the variety of interactions between key players, both at the molecular and cellular levels, and suggest that combined therapies may be the future.

T-cell costimulation pathways (such as CD28/CTLA-4, ICOS-B7RP1, CD70-CD27, CD40-CD154, or OX40-OX40L) may be key players in the pathogenesis of SSc, as suggested by several experimental findings (Boleto et al.). Notably, the blockade of T-cell costimulation with abatacept (CTLA-4-Ig) improved the various manifestations of SSc in pre-clinical animal models (4, 5) and these promising results were corroborated by some preliminary clinical reports (6, 7). Interestingly, abatacept prevented the development of inflammation-driven fibrosis, but demonstrated no efficacy in the treatment of late and non-inflammatory dermal fibrosis (4). Unfortunately, a recent trial (ASSET trial, NCT02161406) did not confirm the promises of abatacept for patients with early diffuse SSc, considering the modified Rodnan skin thickness score (mRSS) at 12 months as primary endpoint (8). Nevertheless, according to this trial, abatacept remains promising when considering some secondary endpoints, *i.e.* the “Health Assessment Questionnaire disability index” (HAQ) and the composite score called “American College of Rheumatology Combined Response Index in diffuse cutaneous Systemic Sclerosis (ACR CRISS)”. These two scores reflect respectively the disability induced by SSc and the probability of improvement in response to treatment in patients with early diffuse cutaneous SSc. Undoubtedly, all these findings illustrate also the complexity and heterogeneity of the disease, as well as the difficulty to monitor SSc and to determine the different components of the disease for a given patient (inflammatory versus dry fibrosis, for example). It also reminds us that encouraging results obtained in animals do not presume a success in humans. So, these -almost- negative results with abatacept suggest that CD28 pathway inhibition alone is insufficient to significantly impact skin disease in patients with early diffuse SSc, and suggest the co-targeting of CD28 with another costimulatory molecule to gain efficacy. For instance, the development of a dual CD28/ICOS antagonist (ALPN-101) may be of interest, since the blockade of both CD28 and ICOS in an acute graft versus host mouse model led to improved survival in ALPN-101-treated mice compared to mice receiving a CD28-CD80/CD86 pathway antagonist (belatacept; CTLA-4-Ig) only (9).

This book also includes a short collection of articles on cell-based therapies in SSc (Maria et al.; Peltzer et al.; Maria et al.; Rozier et al.), and we would like to shortly discuss the progress of knowledge in this field, more precisely on MSC-based therapies. The questions concerning the source and origin of MSC are still under debate, in particular the use of an autologous versus allogeneic approach (23). Concerning MSC and SSc, 12 studies are registered today on the site “ClinicalTrials.gov”, with several distinct methodological approaches. So, we can expect to get answers to many questions in the months and years to come. Another perspective in the field of MSC is represented by their extracellular vesicles (EVs), that function as mediators of intercellular communication and natural transporters of bioactive molecules (proteins, RNA, DNA) to their microenvironment or systemically. Actually, MSC-derived EVs exhibit the main functions of MSC, and thus could constitute an attractive alternative to MSC, *i.e.* not suffering from the potential issues associated with expanded cells. In addition, EVs possess the advantage to be safer (anuclear) and could be stored at room temperature as lyophilized product (10). Recently, our group demonstrated the beneficial effects of EVs obtained from adipose tissue-derived MSC (ASC), both *in vivo* in the HOCl-induced SSc model (11) and *in vitro* in a TGFβ1-induced model of human myofibroblasts, mimicking the characteristics of fibroblasts isolated from SSc patients (12). We also demonstrated that miR-29a-3p (contained in the EVs) plays a pivotal role for the therapeutic effects of ASC, probably by targeting fibrotic, remodeling, apoptotic, and epigenetic processes (11). These findings make even more sense when considering the different key players and mechanisms described in this book. Finally, in parallel with the development of pharmacological molecules, research on cell therapies is also progressing, with interesting therapeutic prospects using the cells or their derived vesicles.

SSc is definitely a complex disease with many key players, which for a long time escaped our understanding and, which we know better, day by day. The challenge of SSc remains still very important, both pathophysiologically and therapeutically. We hope you enjoy reading this book. We hope that it will open up new perspectives for all SSc specialists, and also for all others in their own fields of research.

## Author Contributions

All three authors contributed to the editing of the Research Topic, writing and reviewing of the present Editorial article. All authors contributed to the article and approved the submitted version.

## Conflict of Interest

The authors declare that the research was conducted in the absence of any commercial or financial relationships that could be construed as a potential conflict of interest.

## Publisher’s Note

All claims expressed in this article are solely those of the authors and do not necessarily represent those of their affiliated organizations, or those of the publisher, the editors and the reviewers. Any product that may be evaluated in this article, or claim that may be made by its manufacturer, is not guaranteed or endorsed by the publisher.
